# Neural text generation in regulatory medical writing

**DOI:** 10.3389/fphar.2023.1086913

**Published:** 2023-02-10

**Authors:** Claudia Meyer, Daniel Adkins, Koyena Pal, Ruggero Galici, Augusto Garcia-Agundez, Carsten Eickhoff

**Affiliations:** ^1^ Artificial Intelligence Lab, Brown Center for Biomedical Informatics, Brown University, Providence, RI, United States; ^2^ Khoury College of Computer Sciences, Northeastern University, Boston, MA, United States; ^3^ Pfizer Inc, New York, NY, United States; ^4^ University of Tübingen, Tübingen, Germany

**Keywords:** artificial intelligence-AI, medication guide, drug labeling, natural language generation, abstractive summarization, pointer generator network

## Abstract

**Background:** A steep increase in new drug applications has increased the overhead of writing technical documents such as medication guides. Natural language processing can contribute to reducing this burden.

**Objective:** To generate medication guides from texts that relate to prescription drug labeling information.

**Materials and Methods:** We collected official drug label information from the DailyMed website. We focused on drug labels containing medication guide sections to train and test our model. To construct our training dataset, we aligned “source” text from the document with similar “target” text from the medication guide using three families of alignment techniques: global, manual, and heuristic alignment. The resulting source-target pairs were provided as input to a Pointer Generator Network, an abstractive text summarization model.

**Results:** Global alignment produced the lowest ROUGE scores and relatively poor qualitative results, as running the model frequently resulted in mode collapse. Manual alignment also resulted in mode collapse, albeit higher ROUGE scores than global alignment. Within the family of heuristic alignment approaches, we compared different methods and found BM25-based alignments to produce significantly better summaries (at least 6.8 ROUGE points above the other techniques). This alignment surpassed both the global and manual alignments in terms of ROUGE and qualitative scoring.

**Conclusion:** The results of this study indicate that a heuristic approach to generating inputs for an abstractive summarization model increased ROUGE scores, compared to a global or manual approach when automatically generating biomedical text. Such methods hold the potential to significantly reduce the manual labor burden in medical writing and related disciplines.

## 1 Introduction

The increased discovery and development rate of novel drugs has led to a steep increase in new drug applications submitted to health authorities. This has resulted in an increased volume of medical writing-related technical documents such as protocols, clinical study reports, summaries of drug efficacy and safety, clinical, and non-clinical summaries of pharmacology (ICH, 2022). These technical documents are the source of information for other types of documents such as plain-language summaries and medication guides written for patients, and prescription drug labeling, typically written for healthcare professionals.

Structured Product Labeling (SPL) is a Health Level Seven International standard which defines the content of human prescription drug labeling in an XML format. The “drug labeling” includes all published material accompanying a drug, such as the Prescribing Information, a technical document containing detailed information needed to use the drug safely and effectively. As of Release four of the SPL standard, 22,000 United States Food Drug Administration (FDA) informational product inserts have been encoded according to the standard (FDA, 2022).

SPL documents contain both the content of labeling (i.e., all text, tables and figures) for a product along with additional machine readable information (i.e., drug listing data elements). Drug listing data elements include information about the product (i.e., proprietary and non-proprietary names, ingredients, ingredient strengths, dosage forms, routes of administration, appearance, DEA schedule) and the packaging (i.e., package quantity and type). Large scale publicly accessible databases of SPL data are available, such as Dailymed (FDA, 2019).

Within SPL data, the medication guide is an electronic page or paper handout that accompanies the prescribing information (FDA, 2020). The medication guide does not contain any new information but instead represents a condensed summary of the most important information from all sections of the SPL. The medication guide is recommended by the FDA for certain products in order to provide information regarding known side effects of the product, as well as instructions for safe and effective storage and use. While there are exceptions, a typical medication guide presents information in sections, each headed by a guiding question. Six questions are used most frequently: “What is … ?“, “What is the most important information I should know about … ?“, “Who should not take … ?“, “How should I take … ?“, “What should I avoid while taking … ?“, “What are the possible side effects of … ?”

Medication guides are manually compiled by expert medical writers. Given the considerable length of drug labels, as well as the necessary quality checks, developing these documents is a time-consuming and costly process. An alternative to this approach is to use natural language processing techniques, such as text summarization, to automatically generate medication guides from SPL information.

We conducted a series of studies to test the hypothesis that text can be autogenerated from predefined source documents. Specifically, we present the application of a Pointer-Generator Network (PGN) summarization model to create medication guides from prescription drug labeling information. We compared the effectiveness of various input data selection and model training schemes. We choose to focus on autogenerating medication guides for two main reasons. First, there are thousands of medication guides publicly available. Second, the published medication guides represented an appropriate control for text generated by the algorithm.

## 2 Methods

### 2.1 Data

The dataset was collected from the DailyMed website. This article used the September 2019 edition, providing the official drug labels in XML format. Out of the total 5,357 prescription drug labels 27% of files (1437) provided medication guides. For the remainder of this study we will focus only on these drugs with medication guides present. Medication guides varied in completeness and did not always address all six guiding questions. Table 1 lists the frequency of each question in the dataset alongside the average number of words dedicated to answering the respective question.

**TABLE 1:** Frequency of guiding questions in the dataset.

Question	Frequency (out of 1437)	Answer length (in words)
“What is the most important information I should know about … ?”	87% (1248)	818.34
“What is … ?”	89% (1278)	828.02
“Who should not take … ?”	63% (910)	1019.53
“How should I take … ?”	70% (1002)	1081.59
“What should I avoid while taking … ?”	52% (752)	1343.50
“What are the possible side effects of … ?”	77% (1111)	541.55

### 2.2 Model

Text summarization can be divided into two broad categories: extractive (Nallapati et al., 2017; Zhou et al., 2018; Liu and Lapata, 2019) and abstractive (Rush et al., 2015; Nallapati et al., 2016; See et al., 2017). Given a source document, the goal is to produce a target summary that contains the relevant information in a semantically-condensed form, i.e., using fewer words. Extractive summarization methods produce summaries by selecting and concatenating subsequences of the initial document. For example, a word-level extractive summarization model might summarize the sentence “I am going to take Paracetamol tablets after dinner to reduce side effects of the COVID-19 vaccine, which are muscle aches and chills.” into “I am going to take Paracetamol tablets to reduce muscle aches and chills.” No novel n-grams are produced by the model, as it merely combines pieces of the original text. Similarly, a sentence-level extractive summarization model might summarize a document by selecting and concatenating the introductory sentences of each paragraph.

Abstractive summarization models, on the other hand, create summaries by producing novel n-grams that do not necessarily exist in the source text. The added flexibility can provide richer and more cohesive summaries, tying concepts together in ways that the source text does not. Using the previous word-level example, a strong abstractive summarization model could produce the summary “I will take Paracetamol tablets to reduce side effects of the COVID-19 vaccine”, even though “will” is not in the initial text.

Recurrent neural networks and attentional networks both provide strong frameworks to process sequential data *via* encoder-decoder architectures. Basic Seq2Seq models, which largely follow the encoder-decoder paradigm, have no built-in mechanism for ensuring that the generated information is factually or conceptually correct. For example, an abstractive summarization model might erroneously produce a summary that states “I am going to take two Paracetamol tablets”, when in fact that piece of information was never specified in the source text.

**FIGURE 1:**
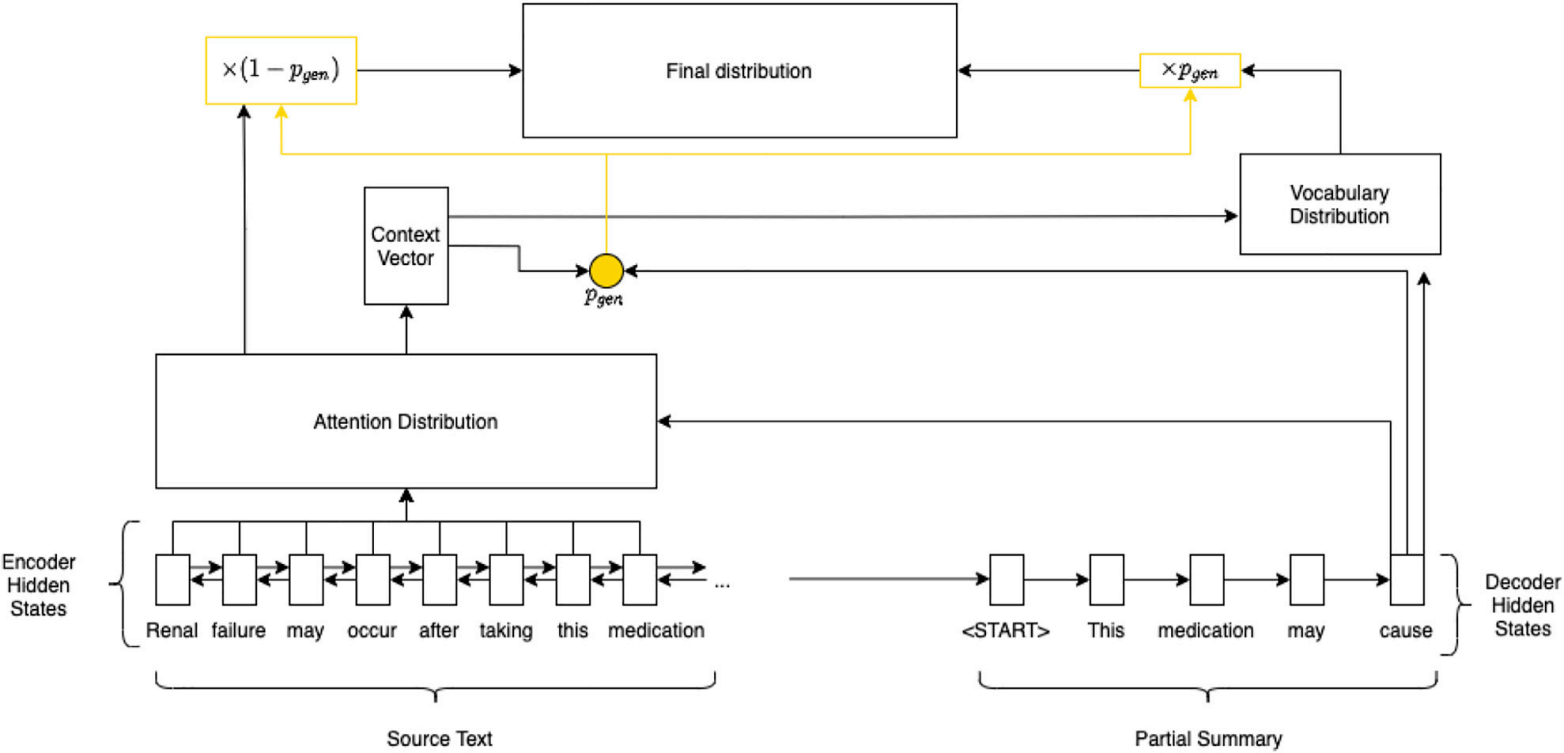
Pointer-generator network.

The PGN (illustrated in Figure 1) aims to solve this information accuracy problem by introducing a pointer mechanism. This allows the model to select and copy tokens from an input text, improving factuality (See et al., 2017).

Nevertheless, long document summarization is still an open challenge due to memory constraints. Particularly notable is the attention mechanism in the Transformer architecture, which requires O (*n* ^2^) attention computations and parameters for an n-token document. In addition, the pointer mechanism requires another O (*n* ^2^) computations and parameters (See et al., 2017).

**FIGURE 2:**
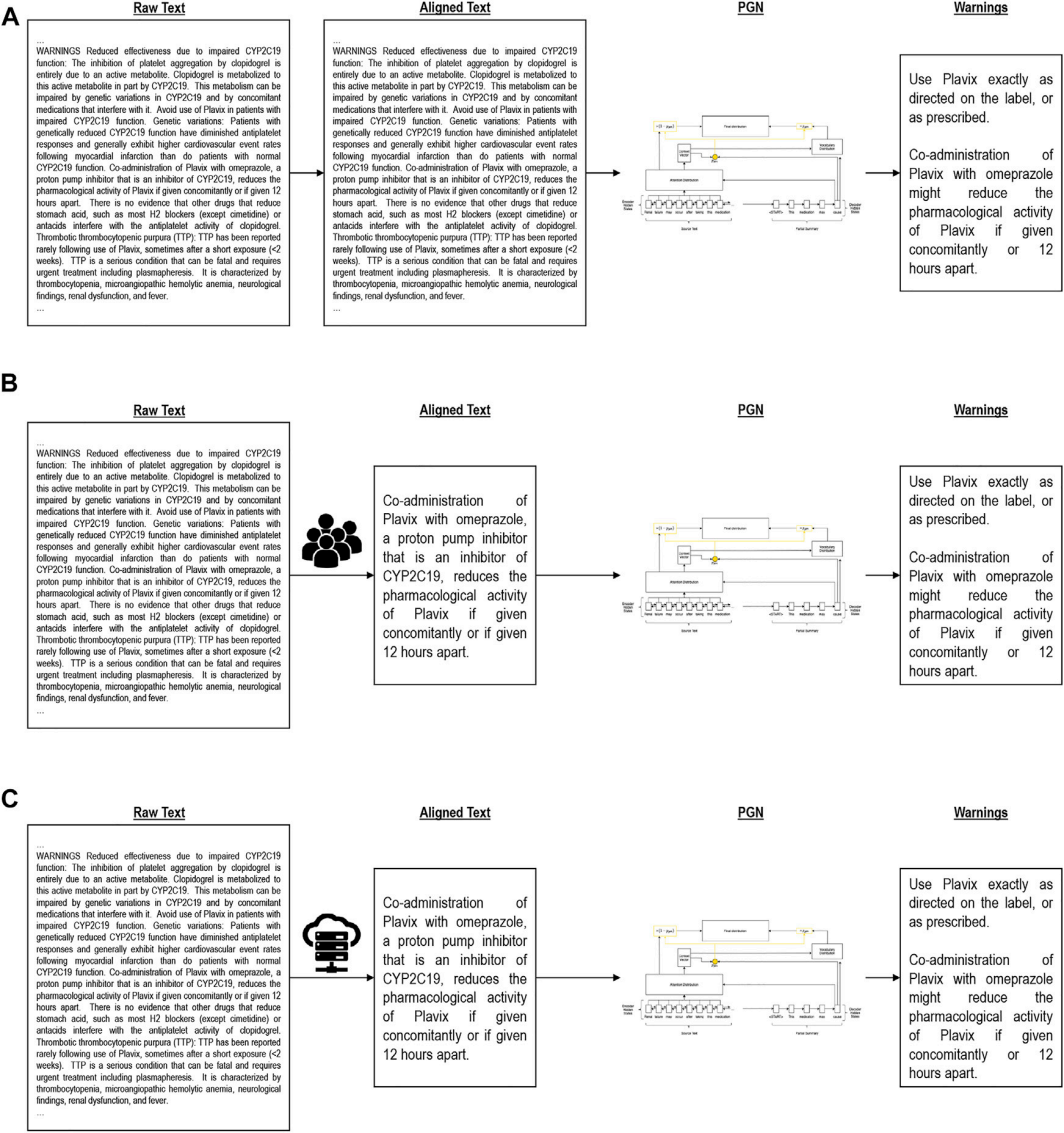
Alignment algorithms. **(A)** is Global alignment, **(B)** is Manual alignment, **(C)** is Heuristic alignment.

We evaluated three training variants to learn to generate medication guides. This includes a one-shot approach using the entire document and entire medication guide, a manual alignment using domain knowledge, and heuristic alignment using a number of similarity approximations (see Figure 2). Table 2 visually illustrates these variants. All experiments were performed on 16GB Quadro RTX 5000 GPUs. Model hyperparameters and other reproducibility information are listed in Supplementary Material.

**TABLE 2:** Comparison of training variants. *: When enough memory is available.

	Memory complexity	Covers full document?	Global attention?	Generalizable?
Global	O (*n* ^2^)	Yes*	Yes	Yes
Manual	O (*n*)	No	No	No
Heuristic	O (*n*)	Yes	No	Yes

#### 2.2.1 Global alignment

The global approach (Figure 2A) takes the entire drug label (without the medication guide) as input and attempts to generate the full medication guide in a single generation step. This baseline model, due to the O (*n* ^2^) complexity of the attention mechanism, quickly reaches a memory bottleneck when scaled to fit longer documents such as drug labels. For relatively long documents, standard GPUs may become insufficient. In these cases, we cap the size of the input document to ensure no memory overflow occurs. Due to this loss of information, the quality of generated medication guides is expected to suffer.

#### 2.2.2 Manual alignment

Using domain knowledge, we manually selected sections of the input drug label corresponding to medication guide questions (Figure 2B). Instead of generating the entire medication guide at once, here we separately generate answers to each guiding question based on the manually identified drug label sections. This condition simulates an idealized setting and represents a headroom analysis of what an effective alignment can achieve.

#### 2.2.3 Heuristic alignment

As a more realistic divide and conquer condition, we split the input document into smaller subsections following the existing section dividers and again generate answers to each medication guide question separately (Figure 2C). Instead of relying on manual alignment, we explored a number of text similarity heuristics to model the similarity of drug label sections (input) to medication guide answers (output). The single subsection with the highest similarity is used for training, and the remainder of the label discarded. Like manual alignment, this allows us to generate smaller input documents that contain key information for summarization. The generalized heuristic alignment process is formalized in Appendix B. We explored five text similarity heuristics for automatic input/output alignment: (1) TF-IDF ‘best match’, (2) TF-IDF ‘average match’, (3) TF-IDF ‘median match’, (4) LSH + Jaccard Similarity, (5) BERT. The technical details of these similarity metrics are discussed in Appendix C.

## 3 Results

The dataset was split into 60% training data, 20% testing, and 20% validation sets. The following results are from model runs on the test sets. Table 3 reports model performance in terms of F1 ROUGE-1, F1 ROUGE-2, and F1 ROUGE-L scores between generated and reference medication guides.

**TABLE 3:** Test set performance.

Method	F1 ROUGE-1	F1 ROUGE-2	F1 ROUGE-L
Global	0.462	0.441	0.43
Manual “Contraindications”	0.63	0.56	0.617
Manual “Interactions”	0.57	0.44	0.55
Manual “Warnings”	0.33	0.21	0.319
LSH Jaccard	50.50	47.20	48.20
BERT	52.0	43.90	48.50
TF-IDF ‘best match’	58.90	52.26	55.0
TF-IDF ‘average match’	60.20	54.20	56.70
TF-IDF ‘median match’	67.0	64.0	64.0

### 3.1 Global alignment

As expected, one-shot summarization of entire medication guides produced both quantitatively and qualitatively poor results. This is likely due to the model’s inability to attend over the entire input drug label, and thus only capturing a limited amount of the text to use for summarization. Furthermore, unlike other methods, there is no guarantee that the available (pre-cutoff) tokens contain salient information for producing summaries.

Qualitative inspection showed frequent mode collapse and often contained extremely repetitive or vague summaries. For example, the following summary or a highly similar version was produced for 21 input documents in a sample of 100:

“The risk of getting an ulcer or bleeding increases with: past history of stomach ulcers, or stomach or intestinal bleeding with use of NSAIDs taking medicines called corticosteroids, anticoagulants, SSRIs, or SNRIs increasing doses of NSAIDs longer use of NSAIDs smoking drinking alcohol older age poor health advanced liver disease bleeding problems past history of stomach ulcers, or stomach or intestinal bleeding with use of NSAIDs taking medicines called corticosteroids, anticoagulants, SSRIs, or SNRIs increasing doses of NSAIDs longer use of NSAIDs smoking drinking alcohol older age poor health advanced liver disease bleeding problems.”

This summary, while producing a decent ROUGE score for many documents, is rather unspecific to any drug and lacks the proper structure of a medication guide.

### 3.2 Manual alignment

We compared manually selected input pairings from three different sections (“Contraindications”, “Interactions”, and “Warnings”). The manually aligned “Contraindications” section demonstrated the best performance overall, with higher ROUGE-1, ROUGE-2, and ROUGE-L scores than the global or any of the other manually aligned sections by at least six ROUGE points (Table 3). However, experiments for all three sections resulted in mode collapse, and qualitatively the generated summaries were consistently poor (Table 4). The model seemed to underperform for “Warnings” in particular, and we suspect this is because the source text tended to be significantly longer in this section.

**TABLE 4:** Manual alignment examples.

Section	Source text	References summary	Generated summary
Contra- indications	Potassium chloride is contraindicated in patients on triamterene and amiloride	Concomitant use with triamterene and amiloride (4,7.1)	Concomitant use with triamterene or amiloride (.4). Concomitant use with triamterene or amiloride (.4). Known hypersensitivity or amiloride (4). Patients with in patients with triamterene or amiloride
Interactions	Concomitant administration with gemfibrozil, a strong inhibitor of CYP2C8, doubled exposure to selexipag and increased exposure to the active metabolite by approximately 11-fold. Concomitant administration of UPTRAVI with strong inhibitors of CYP2C8 (e.g., gemfibrozil) is contraindicated [see Contraindications (4) and Clinical Pharmacology (12.3)]. Concomitant administration of UPTRAVI with clopidogrel, a moderate inhibitor of CYP2C8, had no relevant effect on the exposure to selexipag and increased the exposure to the active metabolite by approximately 2.7-fold [see Clinical Pharmacology (12.3)]. Reduce the dosing of UPTRAVI to once daily in patients on a moderate CYP2C8 inhibitor [see Dosage and Administration (2.4)]. Concomitant administration with an inducer of CYP2C8 and UGT 1A3 and 2B7 enzymes (rifampin) halved exposure to the active metabolite. Increase dose up to twice of UPTRAVI when co-administered with rifampin. Reduce UPTRAVI when rifampin is stopped [see Clinical Pharmacology (12.3)]	Moderate cyp2c8 inhibitors (e.g., clopidogrel, deferasirox and teriflunomide) increase exposure to the active metabolite of uptravi. reduce the dosing of uptravi to once daily (2.4,7.1,12.3). CYP2C8 inducers (e.g., rifampin) decrease exposure to the active metabolite. Increase up to twice the dose of uptravi (7.2, 12.3)	CYP2C8 inhibitors (7.1). Concomitant use of clopidogrel, a cyp2c8, doubled exposure with CYP2C8 (7.3). Moderate CYP2C8 (e.g.): reduced moderate CYP2C8 (rifampin): increased risk of approximately CYP2C8 (7.3)
Warnings	Excessive diuresis may cause potentially symptomatic dehydration, blood volume reduction and hypotension and worsening renal function, including acute renal failure particularly in salt-depleted patients or those taking renin-angiotensin aldosterone inhibitors. Worsening of renal function can also occur with concomitant use of nephrotoxic drugs (e.g., aminoglycosides, cisplatin, and NSAIDs). Monitor volume status and renal function periodically. Torsemide can cause potentially symptomatic hypokalemia, hyponatremia, hypomagnesemia, hypocalcemia, and hypochloremic alkalosis. Treatment with torsemide can cause an increase in blood glucose levels and hyperglycemia. Asymptomatic hyperuricemia can occur and gout may rarely be precipitated. Monitor serum electrolytes and blood glucose periodically. Tinnitus and hearing loss (usually reversible) have been observed with loop diuretics, including torsemide. Higher than recommended doses, severe renal impairment, and hypoproteinemia, appear to increase the risk of ototoxicity	Hypotension and worsening renal function: monitor volume status and renal function periodically (5.1). Electrolyte and metabolic abnormalities: monitor serum electrolytes and blood glucose periodically. (5.2).Ototoxicity (5.3, 7.6)	Symptomatic volume reduction reduction and hypotension, acute renal failure particularly in salt-depleted salt-depleted serum electrolytes of nephrotoxic drugs. (5.1). Hyperglycemia and renal impairment: blood serum electrolytes and blood glucose of nephrotoxic drugs (5.2)

### 3.3 Heuristic alignment

Among automatic alignment techniques, the ‘median match’ TF-IDF approach demonstrated the best performance overall, with higher ROUGE-1, ROUGE-2, and ROUGE-L scores than any of the other heuristic alignment approaches by at least 6.8 ROUGE points (Table 3). The ‘median match’ TF-IDF heuristic also produced the most natural appearing medication guides. In some cases, it was able to generate the exact guide. The best automatic alignment technique even outperformed the manual alignment in terms of ROUGE score. We suspect that this is because the heuristic alignment’s use of text similarity metrics allowed for creating closer source/target matches as input for the PGN. Table 5 shows two examples of reference texts and their generated summaries created with this heuristic.

**TABLE 5:** Heuristic alignment examples.

Example	References text	Generated text
Example 1	Take lamotrigine tablets (chewable, dispersible) exactly as prescribed. Your healthcare provider may change your dose. Do not change your dose without talking to your healthcare provider. Do not stop taking lamotrigine tablets (chewable, dispersible) without talking to your healthcare provider. Stopping lamotrigine tablets (chewable, dispersible) suddenly may cause serious problems. For example, if you have epilepsy and you stop taking lamotrigine tablets (chewable, dispersible) suddenly, you may have seizures that do not stop. Talk with your healthcare provider about how to stop lamotrigine tablets (chewable, dispersible) slowly. Do not take 2 doses at the same time. If you take too much lamotrigine, call your healthcare provider or your local poison control center or go to the nearest hospital emergency room right away. You may not feel the full effect of lamotrigine tablets (chewable, dispersible) for several weeks. If you have epilepsy, tell your healthcare provider if your seizures get worse or if you have any new types of seizures. Lamotrigine tablets (chewable, dispersible) may be swallowed whole, chewed, or mixed in water or fruit juice mixed with water. If the tablets are chewed, drink a small amount of water or fruit juice mixed with water to help in swallowing. To break up lamotrigine tablets (chewable, dispersible), add the tablets to a small amount of liquid (1 teaspoon, or enough to cover the medicine) in a glass or spoon. Wait at least 1 min or until the tablets are completely broken up, mix the solution together, and take the whole amount right away	Take lamotrigine tablets (chewable, dispersible) exactly as prescribed. Your healthcare provider may change your dose. Do not change your dose without talking to your healthcare provider. Do not stop taking lamotrigine tablets without talking to your healthcare provider. Stopping lamotrigine tablets suddenly may cause serious problems. For example, if you have epilepsy and you stop taking lamotrigine tablets suddenly, you may have seizures that do not stop. Talk with your healthcare provider about how to stop lamotrigine tablets slowly
Example 2	Do not stop taking carbamazepine without first talking to your healthcare provider. Stopping carbamazepine suddenly can cause serious problems. Stopping seizure medicine suddenly in a patient who has epilepsy may cause seizures that will not stop (status epilepticus). Take carbamazepine exactly as prescribed. Your healthcare provider will tell you how much carbamazepine to take. Your healthcare provider may change your dose. Do not change your dose of carbamazepine without talking to your healthcare provider. Take carbamazepine with food. Carbamazepine extended - release tablets: do not crush, chew, or break carbamazepine extended - release tablets. Tell your healthcare provider if you can not swallow carbamazepine extended - release tablets whole. Carbamazepine oral suspension: shake the bottle well each time before use. Do not take carbamazepine oral suspension at the same time you take other liquid medicines. If you take too much carbamazepine, call your healthcare provider or local poison control center right away	Do not stop taking carbamazepine without first talking to your healthcare provider. Stopping carbamazepine suddenly can cause serious problems. Stopping seizure medicine suddenly in a patient who has epilepsy may cause seizures that will not stop (status epilepticus). Take carbamazepine exactly as prescribed. Your healthcare provider will tell you how much carbamazepine to take. Your healthcare provider may change your dose. Do not change your dose of carbamazepine without talking to your healthcare provider. Take carbamazepine with food. Carbamazepine extended - release tablets: do not crush, chew, or break carbamazepine extended - release

## 4 Discussion

The main goal of this study was to test whether neural text generation is a viable approach to support the development of regulatory documents. In order to test this hypothesis we used medication guides as source documents because large datasets are publicly available, and they represent an appropriate control for automatically generated text. Our results indicate that the ROUGE scores increased when the model shifted from a global to a heuristic approach. These results are not entirely surprising as the global approach is more complex and, due to text length, prone to GPU memory issues.

These results are relevant for several reasons. First, to the best of our knowledge, this is the first time that an algorithm has been used to generate medication guides with robust accuracy.

Second, we believe that accurate generation of text can improve efficiency in medical writing. For example, medication guides are typically generated manually by medical communication specialists and medical writers who choose specific information from the labeling information mainly based on personal experience.

The results of this study also indicates that approximately only a third of sponsor-generated prescription information packages included medication guides. Furthermore, we found that there was a robust level of inconsistency in the medication guides with respect to addressing the six questions recommended by the FDA.

Overall, this study relied on medication guides to demonstrate feasibility of neural text generation. However, we believe that this use case study can be extended to different types of regulatory documents produced by medical writers. Neural text generation has the potential to greatly improve efficiency in medical writing and accelerate regulatory submissions. Potentially, this approach could also be used to create plain-language summaries and medication guides in different languages. Furthermore, more modern encoder-decoder transformers such as GPT-3 (Brown et al., 2020) may improve the performance of the approach presented here. Hovever, the strength of the PGN is that it allows both abstractive and extractive summarization as needed, so further studies comparing both approaches would be required.

Our study shows several limitations. First, the frequently inconsistent structure of medication guides hampers automated approaches. One such inconsistency is the frequency of the six guiding questions, displayed in Table 1. As shown in that table, each question appears a different number of times in the dataset. By having missing data (i.e., questions not asked and answered), the model has an uneven data distribution of sentences summarizing a certain section. Hence, sections that have more frequent similar questions discussed (i.e., contraindication section), are better summarized sections than those that do not have enough instances of uniformly framed questions (i.e. pediatric use section). As the size of datasets increase, this limitation may be at least partially remediated since question discussions will become more numerous and more data will lead to better document generation.

Second, ROUGE scores for summaries generated using the TF-IDF + Euclidean (“median”) aligned inputs are higher than any scores resulting from a manual alignment of inputs, and the generated summaries are similarly higher quality as determined by human evaluation. Manually aligning the “contributions” sections of the documents produces the next highest ROUGE scores, followed by the TF-IDF + Euclidean (“average”) aligned inputs. However, even with the best-performing median heuristic alignment, the generated summaries are not consistently comprehensive and informative. Though the BERT model does not initially show promising results, future work should include exploration of other semantic similarity metrics to improve input pairings, as the best inputs were aligned entirely by their lexical similarity. Another consideration is that the target summaries from the medication guide were often very short, ranging from as few as 12 tokens to around 200. The source text samples were comparatively lengthy, with as many as 2000 tokens, unlike (See et al., 2017), where the article was truncated to 400 tokens and the summary to 100 tokens for training. Achieving more consistent source and target lengths could possibly help the heuristic alignment and our model’s overall performance.

## 5 Conclusion

In this study, we generated medication guides by training a PGN using SPL from the DailyMed database and applying three different alignment techniques: global, manual, and heuristic. We found that heuristic alignment outperformed the other two methods, and showed potential to automatically generate medication guides to reduce the manual burden in medical writing.

## Data Availability

Publicly available datasets were analyzed in this study. This data can be found here: https://dailymed.nlm.nih.gov/dailymed/spl-resources-all-drug-labels.cfm.
