# Supplementary material for: Neural text generation in regulatory medical writing
Source: Front Pharmacol. 2023 Feb 10;14:1086913. doi: 10.3389/fphar.2023.1086913 (PMC9950092; doi:10.3389/fphar.2023.1086913)
Supplement: Supplementary file 1 [file DataSheet1.pdf]

Supplementary Material

1 SUPPLEMENTARY DATA

1.1 Appendix A: Model Hyperparameters

Our implementation followed Abigail See's original implementation https://github.com/abisee/pointer-generator of the PGN. The hyperparameters used were the default parameters set by that implementation, except for the following, which were manually tuned:

- Max encoder size of 1200 (max_enc_steps)
- Beam size of 4 (beam)
- Minimum decoder size of 200 (min_dec_steps)
- Maximum decoder size of 400 (max_dec_steps)
- Batch size of 8 (batch_size)

1.2 Appendix B: Heuristic Alignment of Document and Subsections

Since both splitting and heuristic calculation can be done in an unsupervised manner for any text, the heuristic alignment method is generalizable to any domain.

```
pairs = [ ]
{d_0, d_1, ... d_k} = split(input document)
{t_0, t_1, ... t_k} = split(target summary)
for each input subsection d_i in {d_0, d_1, ... d_k}:
    best_target_subsection = None;
    best_similarity_score = 0;
    for each target t_i in {t_0, t_1, ... t_k}:
        similarity = heuristic(d_i, t_i)
        if similarity > best:
            best_similarity_score = similarity;
            best_target_subsection = t_i
       pairs.append((d_i, t_i))
return pairs
```

1.3 Appendix C: Summaries of Heuristic Alignment Methods

1.3.1 TF-IDF + L2-Normalized Euclidean: "Best hits"

In the best-hits method, target and source text are represented as TF-IDF vectors and the L2-normalized Euclidean distance is calculated between each pair. For each target text section, the closest source by this distance metric is chosen as the best hit, and this (target, source) pair is returned as a match.

1.3.2 TF-IDF + L2-Normalized Euclidean: Average Contributions

In the average contributions method, target and source text are represented as TF-IDF vectors and the L2-normalized Euclidean distance is calculated between each pair. The scores for each pairing are then

averaged across all drug labels, and a ranked list of possible source matches is generated for each target, based on lowest average distance. To generate matches for each target from each drug label, the target's highest ranking source (from the ranked averages list) available in that drug label is chosen, and this (target, source) pair is returned as a match.

1.3.3 TF-IDF + L2-Normalized Euclidean: Median Contributions

In the median contributions method, target and source text are represented as TF-IDF vectors and the L2-normalized Euclidean distance is calculated between each pair. The scores for each pairing are then collected across all documents and the median is found for each possible (target, source) pair. Based on lowest median distance, a ranked list of possible source matches is generated for each target. To generate matches for each target from each document, the target's highest ranking source (from the ranked medians list) available in that document is chosen, and this (target, source) pair is returned as a match.

1.3.4 LSH Forest / Jaccard Distance

In an attempt to reduce the time taken to find source target pairs across all documents, we explored the concept of MinHash Local Sensitive Hashing (LSH). In this approach, each source text is considered a document, and each document is encoded with the MinHash algorithm. Using the datasketch package's MinHashLSHForest Zhu (2021), we added all of the encoded documents to a bucket or index. At query time, the query itself (which is a target text) is hashed to the same bucket. We then request that best match for the top two candidates (or source documents) with the current target document. Based on this algorithm, the best match is considered to be the one whose encoded hash is closest to the query hash. While this allows for quicker computation, we still need to ensure that the top match is indeed a suitable choice.

To do so, the query results were preprocessed and the algorithm then calculated Jaccard distances between each query result and the target text. The lower the Jaccard distance, the higher the similarity. Therefore, the query result that had lower distance value was chosen as the top match to the target text. This process is repeated for all the target texts in the dataset.

1.3.5 BERT-Cosine

Another approach to speed up the process of pairing source target texts accurately is by using sentence transformer networks that are based on pretrained BERT models. The sentences or documents are first preprocessed and are then encoded by the sentence transformer model. These encoded source texts are then compared with the encoded target texts using cosine similarity. The higher the cosine similarity, the better the match. Hence, the source text that had the highest cosine score becomes the top match to the target text.

REFERENCES

[Dataset] Zhu, E. (2021). Minhash lsh forest. `http://ekzhu.com/datasketch/lshforest.html`. Accessed: 2021-01-24
